# Association of Early Albumin Administration with Clinical Outcomes in Patients Hospitalized with Spontaneous Bacterial Peritonitis: A Propensity-Matched Cohort Study

**DOI:** 10.3390/reports9020176

**Published:** 2026-06-06

**Authors:** Noor Albusta, Mohamed Abdulla, Sara Isa, Rehab Almarzooq

**Affiliations:** 1 Department of Internal Medicine, Lahey Hospital and Medical Center, Burlington, MA 01805, USA; 2 School of Medicine, Royal College of Surgeons in Ireland-Medical University of Bahrain, Adliya P.O. Box 15503, Bahrain; mohdabdulla.md1@gmail.com (M.A.); dr.saraisa0@gmail.com (S.I.); 3 Medical Department, Salmaniya Medical Complex, Manama P.O. Box 12, Bahrain; rmarzooq1@hospitals.gov.bh

**Keywords:** spontaneous bacterial peritonitis, albumin, cirrhosis, acute kidney injury, mortality, propensity score matching, hepatorenal syndrome

## Abstract

**Background/Objectives:** Spontaneous bacterial peritonitis (SBP) is a serious complication of decompensated cirrhosis and is associated with acute kidney injury (AKI), organ failure, and death. Intravenous albumin is recommended in SBP because it reduces renal impairment and mortality, particularly in patients at higher risk of circulatory dysfunction and hepatorenal complications. However, the prognostic impact of early albumin administration on clinical outcomes in hospitalized SBP patients remains incompletely characterized in real-world practice. This study aimed to assess the association between early albumin administration and clinical outcomes in patients hospitalized with SBP compared to those without early albumin. **Methods:** A retrospective cohort study was conducted using the TriNetX US Collaborative Research Network, including adults hospitalized with SBP through February 2026. Patients were divided into those receiving early albumin administration (*n* = 1248) and those without early albumin (*n* = 4932) within 24 h of index SBP diagnosis. Propensity score matching (1:1) balanced cohorts (*n* = 1230 each) for demographics, comorbidities, liver disease severity surrogates, medications, and laboratory values. Relative risks (RR), risk differences (RD), and hazard ratios (HR) were calculated using propensity-matched and Cox proportional hazard models. **Results:** Early albumin administration was associated with significantly lower all-cause mortality (RR 0.620; 95% CI: 0.441–0.871; *p* = 0.005 at 5 days; RR 0.770; 95% CI: 0.651–0.910; *p* = 0.002 at 90 days). Secondary outcomes showed reduced risks for acute kidney injury (RR 0.654; 95% CI: 0.553–0.774; *p* < 0.001 at 5 days; RR 0.798; 95% CI: 0.706–0.903; *p* < 0.001 at 90 days), hepatorenal syndrome–AKI (RR 0.598; 95% CI: 0.445–0.804; *p* < 0.001 at 5 days; RR 0.756; 95% CI: 0.613–0.932; *p* = 0.009 at 90 days), vasopressor requirement (RR 0.633; 95% CI: 0.489–0.820; *p* < 0.001 at 5 days; RR 0.712; 95% CI: 0.572–0.887; *p* = 0.002 at 30 days), and renal replacement therapy (RR 0.533; 95% CI: 0.324–0.878; *p* = 0.011 at 5 days; RR 0.642; 95% CI: 0.442–0.932; *p* = 0.019 at 30 days). Cox models confirmed statistically significant risk reductions for all primary and secondary outcomes, including ICU admission (HR 0.82; 95% CI: 0.73–0.92; *p* = 0.001) and 30-day readmission (HR 0.84; 95% CI: 0.73–0.97; *p* = 0.015). Associations were strongest in the early period and attenuated over time. **Conclusions:** Early albumin administration was associated with reduced risks of mortality, AKI, HRS-AKI, and hemodynamic instability in patients hospitalized with SBP, with attenuation over time. These findings support timely implementation of guideline-concordant albumin therapy, although residual confounding cannot be excluded.

## 1. Introduction

Spontaneous bacterial peritonitis is one of the most important infectious complications of decompensated cirrhosis and carries substantial risks of kidney injury, acute-on-chronic decompensation, and death [1,2]. Albumin has an established role in SBP management because plasma volume expansion may prevent circulatory dysfunction and reduce renal impairment [3,4,5]. The landmark randomized trial by Sort et al. demonstrated that albumin added to antibiotics reduced renal impairment and mortality compared with antibiotics alone, and major liver society guidance continues to recommend albumin in this setting [3,4,5,6]. Current guidance commonly supports albumin at 1.5 g/kg on day 1 and 1 g/kg on day 3 alongside appropriate antibiotics [3,4,6].

Although albumin is guideline-supported, real-world practice varies considerably, including whether albumin is given promptly after diagnosis [7,8,9,10]. Delayed treatment may reduce the benefit of albumin, especially in patients at risk for AKI, hepatorenal syndrome–AKI (HRS-AKI), or hemodynamic deterioration [11,12,13]. Recent real-world work also shows continued interest in how albumin timing and dosing affect outcomes in SBP [7,8,9,10]. The need for timely albumin in SBP serves as both a therapeutic intervention for circulatory dysfunction and a potential marker of protocolized, high-quality care. Understanding its prognostic role, whether causal or reflective of illness recognition and care delivery, is critical. Albumin may improve outcomes through effective arterial volume expansion and modulation of SBP-related circulatory dysfunction, although early administration may also reflect earlier clinical recognition and more comprehensive care [3,4,5,6,7,8,9,10,11,12,13].

Prior studies linking albumin to improved outcomes in SBP are limited by small sample sizes, single-center designs, and inconsistent definitions of albumin timing and dosing, reducing generalizability [7,8,9,10,14,15,16]. Yet, high-grade, practical evidence across diverse populations with variation in comorbidities and geographic representation remains limited in evaluating the independent prognostic impact of early albumin administration [7,8,9,10,14,15,16].

To address this knowledge gap, we conducted a multicenter retrospective cohort study using the TriNetX US Collaborative Research Network, which connects electronic health records from hospitals, health systems, and research institutions across the United States. This study aims to compare outcomes in patients hospitalized with SBP with and without early albumin administration. It mainly focuses on mortality, renal complications, hemodynamic instability, and rehospitalization. This study provides apt evidence to inform albumin administration practices, improve risk stratification, and refine management strategies in contemporary SBP care.

## 2. Methods

### 2.1. Data Source and Study Design

This study was a retrospective cohort analysis using the TriNetX US Collaborative Research Network (TriNetX, LLC, Cambridge, MA, USA), a large federated database of de-identified electronic health records from multiple healthcare organizations across the United States. The platform includes patient demographics, diagnoses, medications, laboratory results, procedures, and clinical outcomes. Institutional review board approval was not required because the study used de-identified, HIPAA-compliant data.

### 2.2. Study Population

For the selected study population, we identified all adults (≥18 years) hospitalized with spontaneous bacterial peritonitis using ICD-10 diagnosis codes and supporting clinical and laboratory criteria available within the platform. Patients were divided into two groups: those who received intravenous albumin within 24 h of the index SBP diagnosis (early albumin group) and those who did not receive albumin within 24 h (no early albumin group). We selected this window to capture the acute phase of SBP in which circulatory dysfunction and renal injury are most likely to develop.

We acknowledge that defining early albumin administration within a 24-h window may encompass heterogeneous clinical scenarios, as some patients may have received albumin for indications other than SBP alone, while others may have had albumin initiated at varying points within the 24-h period. To address this potential heterogeneity, a restricted subgroup analysis excluding patients with septic shock at presentation was performed. Patients were excluded if they were younger than 18 years, had missing demographic data, had secondary peritonitis or an intra-abdominal surgical source of infection, had peritoneal dialysis-associated peritonitis, had undergone prior liver transplantation, had advanced shock present before the index SBP diagnosis, or had end-stage renal disease on chronic dialysis at baseline. These exclusion criteria were applied uniformly to both the early albumin and no early albumin cohorts.

### 2.3. Baseline Characteristics

For each selected patient, we collected information regarding age, sex, race/ethnicity, and comorbidities such as type 2 diabetes mellitus, chronic kidney disease, heart failure, hypertension, hepatic encephalopathy, variceal bleeding history, and ascites. In addition, laboratory values were obtained including baseline serum creatinine, total bilirubin, INR, serum albumin, and sodium. MELD-Na surrogate variables were collected where available. Medication use was recorded including antibiotics (ceftriaxone or cefotaxime), vasopressors, diuretics, lactulose, rifaximin, and midodrine. All baseline variables were included in propensity score matching.

### 2.4. Study Endpoints

The primary outcome in this study was all-cause mortality, assessed at 5 days, 30 days, and 90 days after the index SBP diagnosis.

Secondary outcomes included acute kidney injury (AKI), hepatorenal syndrome–AKI (HRS-AKI), ICU admission, vasopressor requirement or hemodynamic instability, need for renal replacement therapy, 30-day hospital readmission, and length of stay. These outcomes were identified using validated diagnosis and procedure codes available within TriNetX.

### 2.5. Statistical Analysis

Baseline characteristics were compared between groups using chi-square tests for categorical variables and Student’s *t*-tests for continuous variables. To account for baseline differences, we performed 1:1 propensity score matching (PSM) using the nearest-neighbor approach with a caliper of 0.01. Variables included in the matching model were demographics, comorbidities, laboratory values, and medication use. Balance between groups was assessed with standardized mean differences (SMD), with values < 0.1 considered acceptable. For each outcome, we calculated relative risks (RR) and risk differences (RD) with 95% confidence intervals (CI) at predefined follow-up intervals of 5 days, 30 days, and 90 days. To address potential immortal-time bias and the time-dependent nature of the exposure, we performed a multivariable Cox proportional hazards regression analysis. This model calculates hazard ratios (HR) for the primary and secondary outcomes, adjusting for the same comprehensive set of covariates used in the PSM. This “double-robust” approach ensures that the impact of early albumin is evaluated as a time-to-event variable, mitigating the limitations of fixed-point analysis. To address residual confounding by indication, we performed restricted analyses excluding patients with septic shock at presentation, with new propensity-based adjustment and Cox models in this complication-free subgroup. Statistical significance was defined as a two-sided *p*-value < 0.05. All analyses were conducted within the TriNetX platform.

## 3. Results

### 3.1. Patient Selection

**Figure 1:**
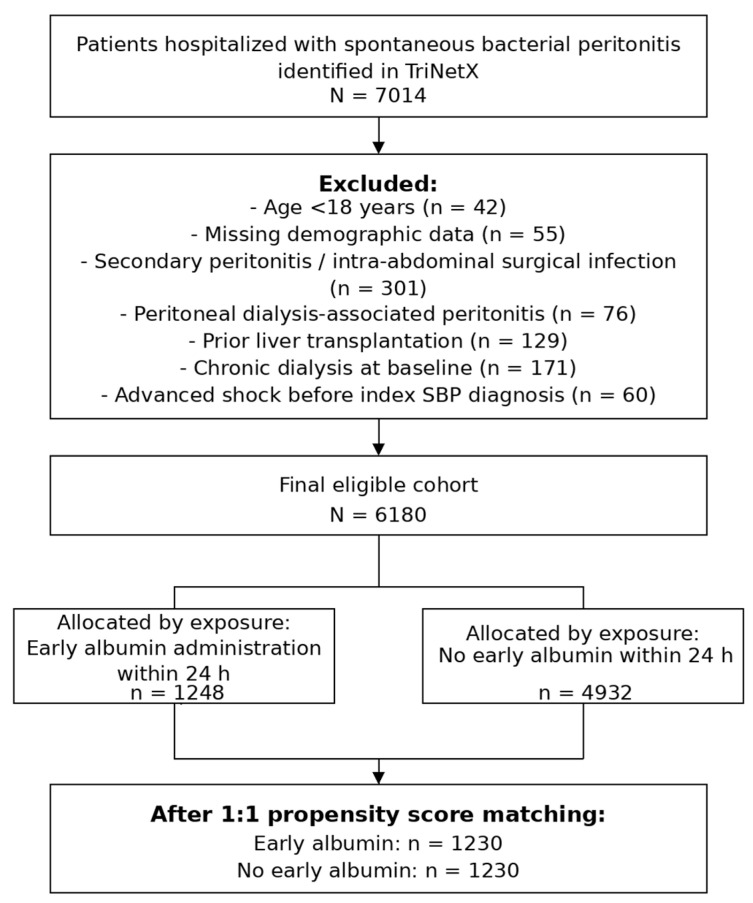
Flowchart of patient selection.

A total of 7014 patients hospitalized with spontaneous bacterial peritonitis were identified on the TriNetX US Collaborative Research Network. After applying exclusion criteria (age < 18 years, n = 42; missing demographic data, n = 55; secondary peritonitis or intra-abdominal surgical infection, n = 301; peritoneal dialysis-associated peritonitis, n = 76; prior liver transplantation, n = 129; chronic dialysis at baseline, n = 171; advanced shock before index SBP diagnosis, n = 60), a final eligible cohort of 6180 patients remained. Of these, 1248 (20.2%) received early albumin administration and 4932 (79.8%) did not. After 1:1 propensity score matching, 1230 patients remained in each group (Figure 1). This matched cohort was consistently used for all outcome analyses across follow-up intervals of 5 days, 30 days, and 90 days. All analyses were repeated after excluding patients with septic shock at presentation.

### 3.2. Population Characteristics

Before PSM, patients in the early albumin group were younger (58.7 ± 11.9 vs. 60.9 ± 12.8, *p* < 0.001) and had higher proportions of females (37.0% vs. 33.9%, *p* = 0.038). Both cohorts were predominantly White (64.2% vs. 63.3%). The early albumin group showed higher prevalence of type 2 diabetes (38.3% vs. 34.2%, *p* = 0.007), chronic kidney disease (29.3% vs. 24.2%, *p* < 0.001), hepatic encephalopathy (41.7% vs. 34.9%, *p* < 0.001), and variceal bleeding history (18.6% vs. 15.1%, *p* = 0.003). Laboratory values differed, with higher baseline creatinine (1.21 ± 0.48 vs. 1.13 ± 0.44, *p* < 0.001), higher total bilirubin (4.0 ± 3.1 vs. 3.4 ± 2.8, *p* < 0.001), higher INR (1.79 ± 0.54 vs. 1.67 ± 0.49, *p* < 0.001), lower serum albumin (2.33 ± 0.42 vs. 2.41 ± 0.46, *p* < 0.001), and lower sodium (131.4 ± 5.5 vs. 132.6 ± 5.2, *p* < 0.001) in the early albumin group, consistent with more advanced liver disease. PSM effectively balanced these differences (all SMD < 0.1 post-PSM; Table 1).

### 3.3. Primary Outcomes

The primary outcome was assessed at 5 days, 30 days, and 90 days. Table 2 presents the primary and secondary outcomes of SBP patients with and without early albumin after PSM. At 5 days, all-cause mortality was significantly lower in the early albumin group (RR 0.620; 95% CI: 0.441–0.871; *p* = 0.005; RD −0.024; 95% CI: −0.040 to −0.009). This association persisted at 30 days (RR 0.699; 95% CI: 0.563–0.869; *p* = 0.001; RD −0.042; 95% CI: −0.067 to −0.016) and remained significant at 90 days though attenuated (RR 0.770; 95% CI: 0.651–0.910; *p* = 0.002; RD −0.048; 95% CI: −0.077 to −0.018). At 5 days, early albumin was significantly associated with reduced risk of vasopressor requirement (RR 0.633; 95% CI: 0.489–0.820; *p* < 0.001), which persisted at 30 days (RR 0.712; 95% CI: 0.572–0.887; *p* = 0.002). ICU admission was also significantly lower at 5 days (RR 0.791; 95% CI: 0.679–0.920; *p* = 0.002) and 30 days (RR 0.833; 95% CI: 0.727–0.954; *p* = 0.009). AKI was significantly reduced at all timepoints, with the strongest association at 5 days (RR 0.654; 95% CI: 0.553–0.774; *p* < 0.001) and persistent benefit at 90 days (RR 0.798; 95% CI: 0.706–0.903; *p* < 0.001). HRS-AKI followed a similar pattern (RR 0.598; 95% CI: 0.445–0.804; *p* < 0.001 at 5 days; RR 0.756; 95% CI: 0.613–0.932; *p* = 0.009 at 90 days). Renal replacement therapy was significantly lower at 5 days (RR 0.533; 95% CI: 0.324–0.878; *p* = 0.011) and 30 days (RR 0.642; 95% CI: 0.442–0.932; *p* = 0.019). Thirty-day readmission was also reduced (RR 0.838; 95% CI: 0.717–0.980; *p* = 0.028). All outcome associations attenuated over time (Table 2).

### 3.4. Length of Stay and Continuous Outcomes

Early albumin administration was associated with a significantly shorter length of stay (8.7 ± 5.6 vs. 10.1 ± 6.3 days; mean difference −1.4; 95% CI: −1.9 to −0.9; *p* < 0.001). Peak creatinine during admission was lower in the early albumin group (1.64 ± 0.91 vs. 1.92 ± 1.05 mg/dL; mean difference −0.28; 95% CI: −0.36 to −0.20; *p* < 0.001), and the change in creatinine from baseline to peak was also smaller (+0.39 ± 0.52 vs. +0.62 ± 0.67 mg/dL; mean difference −0.23; 95% CI: −0.28 to −0.18; *p* < 0.001). Among patients admitted to the ICU, ICU length of stay was shorter in the early albumin group (4.2 ± 3.1 vs. 5.1 ± 3.8 days; mean difference −0.9; 95% CI: −1.4 to −0.4; *p* = 0.001) (Table 3).

### 3.5. Cox Proportional Hazard Model

All-cause mortality was associated with a significantly lower hazard in the early albumin group (HR 0.72; 95% CI: 0.61–0.85; *p* < 0.001). Significant risk reductions were seen for acute kidney injury (HR 0.69; 95% CI: 0.61–0.78; *p* < 0.001), HRS-AKI (HR 0.73; 95% CI: 0.61–0.88; *p* < 0.001), and vasopressor requirement (HR 0.66; 95% CI: 0.55–0.81; *p* < 0.001). ICU admission (HR 0.82; 95% CI: 0.73–0.92; *p* = 0.001), renal replacement therapy (HR 0.64; 95% CI: 0.45–0.92; *p* = 0.010), and 30-day readmission (HR 0.84; 95% CI: 0.73–0.97; *p* = 0.015) were also significantly reduced (Table 4).

### 3.6. Restricted Analyses

To address residual confounding by indication, analyses were repeated in a restricted subgroup excluding patients with septic shock at presentation. After new PSM in this subgroup (n = 980 per group), baseline characteristics were balanced (SMD < 0.1). Associations persisted but were attenuated compared to the full cohort. All-cause mortality at 30 days remained significantly lower (RR 0.70; 95% CI: 0.53–0.92; HR 0.76; 95% CI: 0.60–0.95; *p* = 0.017). AKI at 5 days was significantly reduced (RR 0.69; 95% CI: 0.57–0.83; HR 0.72; 95% CI: 0.60–0.86; *p* < 0.001). HRS-AKI at 30 days remained significant (RR 0.71; 95% CI: 0.53–0.95; HR 0.75; 95% CI: 0.58–0.98; *p* = 0.031). Vasopressor requirement at 5 days was also significantly lower (RR 0.64; 95% CI: 0.46–0.87; HR 0.68; 95% CI: 0.51–0.90; *p* = 0.006) (Table 5).

## 4. Discussion

### 4.1. Principal Findings

In our cohort of over 6000 patients hospitalized with SBP, those who received early albumin administration had lower risks of death, renal complications, and hemodynamic deterioration, particularly in the days following diagnosis. These risks remained evident at 90 days, though the strength of the association declined over time. Prior studies have reported similar findings: the landmark randomized trial by Sort et al. demonstrated that albumin added to antibiotics reduced renal impairment and mortality, and the me-ta-analysis by Salerno et al. confirmed these benefits across pooled randomized data [17,18]. Major liver society guidelines, including those from the AASLD and AGA, continue to recommend albumin at 1.5 g/kg on day 1 and 1 g/kg on day 3 alongside appropriate antibiotics [19,20].

### 4.2. Biological Plausibility and Clinical Context

The strongest associations appeared in the early period, which is biologically plausible given that albumin in SBP is intended to counter effective arterial hypovolemia and prevent renal dysfunction during the acute inflammatory phase [19,20,21,22]. Since AKI and circulatory dysfunction often develop early in SBP, delayed albumin may miss the window in which hemodynamic support is most protective [23,24,25]. This temporal pattern is consistent with the pathophysiology of SBP-related circulatory dysfunction, in which bacterial translocation triggers a cascade of splanchnic vasodilation, reduced effective arterial blood volume, and activation of the ren-in-angiotensin-aldosterone system that can precipitate hepatorenal physiology within hours to days of infection onset [21,22,26].

In SBP, bacterial translocation and systemic inflammation worsen the already vasodilated circulatory state of advanced cirrhosis. This leads to reduced effective arterial blood volume, activation of the renin–angiotensin–aldosterone and sympathetic nervous systems, renal vasoconstriction, and increased risk of AKI or HRS-AKI. Albumin may improve outcomes not only by expanding plasma volume and supporting renal perfusion, but also through non-oncotic effects, including binding of inflammatory mediators and bacterial products, antioxidant activity, endothelial stabilization, and modulation of systemic inflammation. Therefore, the stronger early association observed in our study is biologically plausible, as albumin is likely most beneficial before irreversible renal or hemodynamic deterioration has occurred [21,22,26].

Our Cox models showed consistent associations across all outcomes, suggesting that the protective signal persists even after rigorous adjustment for confounders. After adjustment, the associations with mortality, AKI, and HRS-AKI remained statistically significant, indicating a persistent signal even after accounting for baseline differences. The finding that only 20.2% of patients received early albumin is concerning from a quality standpoint and consistent with known real-world practice gaps in guideline-concordant SBP management [27,28].

### 4.3. Care Quality and Interpretation

Adding to the complexity, patients who receive early albumin may be in centers with more protocolized hepatology care, earlier paracentesis, faster antibiotic initiation, and more experienced teams [23,24,27,28]. Thus, early albumin may serve as both a therapeutic intervention and a marker of higher-quality, more protocolized care. This dual role makes it difficult to isolate the independent effect of albumin timing from the broader care environment. Consistent with this concept, the restricted subgroup analysis excluding patients with septic shock showed persistent but attenuated associations, supporting the interpretation that early albumin reflects a combination of direct therapeutic benefit and overall care quality rather than acting as an isolated causal factor.

Albumin may contribute to improved outcomes through multiple mechanisms beyond simple volume expansion, including binding and neutralization of pathogen-associated molecular patterns, antioxidant activity, endothelial stabilization, and modulation of systemic inflammation [21,22,26]. In practice, albumin administration often reflects the severity of the clinical presentation while also providing direct hemodynamic and renal protection. This supports the use of timely, guideline-concordant albumin dosing in SBP [17,18,19,20]. The SCCM issued a strong recommendation for albumin at SBP diagnosis even without obvious need for volume resuscitation, and the AGA Clinical Practice Update emphasizes the importance of the specific 1.5 g/kg plus 1 g/kg dosing regimen [19,20].

Early albumin administration may also function as a marker of standardized, high-quality SBP care rather than an isolated therapeutic exposure. Patients who received albumin promptly may have also been more likely to undergo early diagnostic paracentesis, timely antibiotic administration, hepatology consultation, renal monitoring, and protocolized inpatient management [19,20,24,27,28]. Therefore, the observed associations likely reflect a combination of albumin’s direct physiological effects and broader guide-line-concordant care delivery. This distinction is important, as our observational design cannot fully separate the independent biological effect of albumin from the effect of higher-quality care [19,20,27,28].

Although albumin is guideline-supported in SBP, early administration may carry potential risks, particularly in patients with cardiac dysfunction, renal impairment, or baseline volume overload. Possible adverse effects include pulmonary edema, volume overload, and worsening heart failure [19,20,21,22]. In this database-based study, these adverse events could not be reliably adjudicated or directly attributed to albumin exposure. However, early albumin was not associated with worse resource-related outcomes and was instead associated with lower ICU admission, renal replacement therapy, vasopressor requirement, and shorter length of stay. Future prospective studies should evaluate both efficacy and albumin-related safety outcomes, including pulmonary edema, oxygen requirement, and heart failure exacerbation [21,22].

### 4.4. Limitations

Our study has important limitations inherent to registry-based analyses. First, while we adjusted for baseline comorbidities and liver disease severity surrogates, the administrative nature of the dataset limits our ability to capture granular clinical details, such as ascitic fluid polymorphonuclear cell count trends, culture data, exact timing of paracentesis relative to antibiotics, or whether the full albumin dosing protocol (1.5 g/kg on day 1 and 1 g/kg on day 3) was completed [2,3,4,9,10,11,12]. Second, although we employed Cox proportional hazards modeling to account for time-to-event data, residual confounding by indication, where early albumin serves as a marker for protocolized care or earlier clinical recognition rather than a direct cause of benefit, cannot be entirely ruled out [9,10,11,12,28]. Our exposure definition may have introduced heterogeneity, as some patients may have received albumin for indications other than SBP alone, which could affect the interpretation of the exposure. Despite propensity matching, residual confounding by indication and reverse causation remain likely, as early albumin administration is often a consequence of more severe presentations prompting faster clinical action. Our restricted subgroup analyses excluding patients with septic shock showed consistent but attenuated associations, supporting early albumin as potentially reflecting both direct benefit and overall care quality. TriNetX lacks granular clinical variables such as full MELD-Na scores, ascitic fluid analysis trends, exact antibiotic timing, repeat paracentesis data, and albumin dose completion. This introduces residual confounding that cannot be fully addressed by PSM. Third, albumin-related adverse events, including pulmonary edema, volume overload, and worsening heart failure, could not be reliably assessed or attributed using the available database variables. Finally, we lacked data on the exact albumin dose administered per kilogram of body weight, preventing a dose–response analysis.

## 5. Conclusions

This large-scale propensity-matched analysis demonstrates that early albumin administration is associated with lower risks of all-cause mortality, acute kidney injury, hepatorenal syndrome–AKI, and hemodynamic instability in patients hospitalized with spontaneous bacterial peritonitis. These associations may reflect albumin as both a direct therapeutic intervention and a marker of timely, guideline-concordant care, pending further causal studies. By utilizing time-to-event Cox regression modeling, we demonstrated that the reduced risk associated with early albumin persists even after rigorous adjustment for confounders, particularly in the immediate post-diagnosis phase. While our restricted analyses suggest that the benefit may reflect a combination of direct albumin effect and overall care quality, this interpretation is hypothesis generating, as formal mediation analysis is needed to confirm any causal pathways. These findings support the timely implementation of guideline-concordant albumin therapy in SBP and emphasize the importance of protocolized care delivery, early paracentesis, and prompt antibiotic initiation to optimize outcomes in this vulnerable population.

## Figures and Tables

**Table 1:** Baseline demographic and clinical characteristics before and after propensity score matching.

Characteristic	Before PSM Early Albumin (N = 1248)	Before PSM No Early Albumin (N = 4932)	*p* Value	SMD	After PSM Early Albumin (N = 1230)	After PSM No Early Albumin (N = 1230)	*p* Value	SMD
**Age, years**	58.7 ± 11.9	60.9 ± 12.8	<0.001	0.178	58.8 ± 11.9	59.0 ± 12.1	0.721	0.016
**Female**	462 (37.0%)	1673 (33.9%)	0.038	0.064	456 (37.1%)	449 (36.5%)	0.755	0.012
**White**	801 (64.2%)	3121 (63.3%)	0.569	0.019	789 (64.1%)	792 (64.4%)	0.901	0.006
**Black**	181 (14.5%)	694 (14.1%)	0.719	0.011	179 (14.6%)	176 (14.3%)	0.822	0.008
**Hispanic**	201 (16.1%)	763 (15.5%)	0.580	0.017	198 (16.1%)	194 (15.8%)	0.834	0.008
**Type 2 diabetes**	478 (38.3%)	1685 (34.2%)	0.007	0.086	468 (38.0%)	464 (37.7%)	0.883	0.006
**CKD**	366 (29.3%)	1192 (24.2%)	<0.001	0.117	355 (28.9%)	349 (28.4%)	0.789	0.011
**Hepatic encephalopathy**	521 (41.7%)	1722 (34.9%)	<0.001	0.140	510 (41.5%)	503 (40.9%)	0.766	0.012
**Variceal bleeding history**	232 (18.6%)	744 (15.1%)	0.003	0.094	225 (18.3%)	220 (17.9%)	0.807	0.010
**Baseline creatinine, mg/dL**	1.21 ± 0.48	1.13 ± 0.44	<0.001	0.174	1.20 ± 0.47	1.19 ± 0.46	0.662	0.020
**Sodium, mmol/L**	131.4 ± 5.5	132.6 ± 5.2	<0.001	0.224	131.5 ± 5.5	131.6 ± 5.3	0.789	0.018
**Total bilirubin, mg/dL**	4.0 ± 3.1	3.4 ± 2.8	<0.001	0.204	3.9 ± 3.0	3.8 ± 2.9	0.548	0.030
**INR**	1.79 ± 0.54	1.67 ± 0.49	<0.001	0.232	1.78 ± 0.53	1.77 ± 0.52	0.713	0.018
**Serum albumin, g/dL**	2.33 ± 0.42	2.41 ± 0.46	<0.001	0.180	2.34 ± 0.42	2.35 ± 0.44	0.676	0.022
**Ceftriaxone or cefotaxime use**	1010 (80.9%)	3702 (75.1%)	<0.001	0.142	997 (81.1%)	991 (80.6%)	0.776	0.012
**Diuretic use**	714 (57.2%)	2554 (51.8%)	0.001	0.108	705 (57.3%)	696 (56.6%)	0.734	0.014

Abbreviations: CKD = chronic kidney disease; INR = international normalized ratio; PSM = propensity score matching; SMD = standardized mean difference. Continuous variables are presented as the mean ± standard deviation and categorical variables as n (%).

**Table 2:** Relative risks and risk differences for clinical outcomes after propensity score matching.

Outcome	Timepoint	Early Albumin (n/N)	No Early Albumin (n/N)	RD (95% CI)	RR (95% CI)	*p* Value
**All-cause mortality**	5 Days	49/1230	79/1230	−0.024 (−0.040, −0.009)	0.620 (0.441–0.871)	0.005
**30 Days**	121/1230	173/1230	−0.042 (−0.067, −0.016)	0.699 (0.563–0.869)	0.001	
**90 Days**	198/1230	257/1230	−0.048 (−0.077, −0.018)	0.770 (0.651–0.910)	0.002	
**Acute kidney injury**	5 Days	176/1230	269/1230	−0.076 (−0.108, −0.044)	0.654 (0.553–0.774)	<0.001
**30 Days**	242/1230	331/1230	−0.072 (−0.107, −0.037)	0.731 (0.634–0.842)	<0.001	
**90 Days**	296/1230	371/1230	−0.061 (−0.098, −0.024)	0.798 (0.706–0.903)	<0.001	
**HRS-AKI**	5 Days	67/1230	112/1230	−0.037 (−0.057, −0.016)	0.598 (0.445–0.804)	<0.001
**30 Days**	104/1230	149/1230	−0.037 (−0.062, −0.013)	0.698 (0.550–0.886)	0.003	
**90 Days**	133/1230	176/1230	−0.035 (−0.062, −0.007)	0.756 (0.613–0.932)	0.009	
**ICU admission**	5 Days	219/1230	277/1230	−0.047 (−0.079, −0.015)	0.791 (0.679–0.920)	0.002
**30 Days**	265/1230	318/1230	−0.043 (−0.077, −0.008)	0.833 (0.727–0.954)	0.009	
**Vasopressor requirement/hemodynamic instability**	5 Days	88/1230	139/1230	−0.041 (−0.062, −0.020)	0.633 (0.489–0.820)	<0.001
**30 Days**	121/1230	170/1230	−0.040 (−0.065, −0.015)	0.712 (0.572–0.887)	0.002	
**Renal replacement therapy**	5 Days	24/1230	45/1230	−0.017 (−0.029, −0.004)	0.533 (0.324–0.878)	0.011
**30 Days**	43/1230	67/1230	−0.020 (−0.035, −0.004)	0.642 (0.442–0.932)	0.019	
**30-day readmission**	30 Days	227/1230	271/1230	−0.036 (−0.068, −0.005)	0.838 (0.717–0.980)	0.028

Abbreviations: AKI = acute kidney injury; CI = confidence interval; HRS-AKI = hepatorenal syndrome–acute kidney injury; ICU = intensive care unit; RD = risk difference; RR = relative risk. Outcomes are shown after propensity score matching.

**Table 3:** Length of stay and selected continuous outcomes after propensity score matching.

Outcome	Early Albumin (N = 1230)	No Early Albumin (N = 1230)	Mean Difference (95% CI)	*p* Value
**Length of stay, days**	8.7 ± 5.6	10.1 ± 6.3	−1.4 (−1.9 to −0.9)	<0.001
**Peak creatinine during admission, mg/dL**	1.64 ± 0.91	1.92 ± 1.05	−0.28 (−0.36 to −0.20)	<0.001
**Change in creatinine from baseline to peak, mg/dL**	+0.39 ± 0.52	+0.62 ± 0.67	−0.23 (−0.28 to −0.18)	<0.001
**ICU length of stay among ICU patients, days**	4.2 ± 3.1	5.1 ± 3.8	−0.9 (−1.4 to −0.4)	0.001

Abbreviations: CI = confidence interval; ICU = intensive care unit. Continuous variables are presented as the mean ± standard deviation.

**Table 4:** Adjusted hazard ratios for outcomes associated with early albumin administration.

Outcome	HR	Coefficient	SE	z	*p* Value	95% CI
**All-cause mortality**	0.72	−0.329	0.084	−3.92	<0.001	0.61–0.85
**Acute kidney injury**	0.69	−0.371	0.061	−6.08	<0.001	0.61–0.78
**HRS-AKI**	0.73	−0.315	0.088	−3.58	<0.001	0.61–0.88
**ICU admission**	0.82	−0.198	0.057	−3.47	0.001	0.73–0.92
**Vasopressor requirement**	0.66	−0.416	0.091	−4.57	<0.001	0.55–0.81
**Renal replacement therapy**	0.64	−0.446	0.173	−2.58	0.010	0.45–0.92
**30-day readmission**	0.84	−0.174	0.072	−2.42	0.015	0.73–0.97

Abbreviations: CI = confidence interval; HR = hazard ratio; SE = standard error; ICU = intensive care unit; HRS-AKI = hepatorenal syndrome–acute kidney injury.

**Table 5:** Restricted subgroup analysis excluding patients with septic shock at presentation (post-PSM, n = 980 per group).

Outcome	Timepoint	Events Early Albumin	Events No Early Albumin	RR (95% CI)	HR (95% CI)	*p* Value
**All-cause mortality**	30 Days	78	112	0.70 (0.53–0.92)	0.76 (0.60–0.95)	0.017
**AKI**	5 Days	129	186	0.69 (0.57–0.83)	0.72 (0.60–0.86)	<0.001
**HRS-AKI**	30 Days	72	101	0.71 (0.53–0.95)	0.75 (0.58–0.98)	0.031
**Vasopressor requirement**	5 Days	59	92	0.64 (0.46–0.87)	0.68 (0.51–0.90)	0.006

Abbreviations: AKI = acute kidney injury; CI = confidence interval; HR = hazard ratio; HRS-AKI = hepatorenal syndrome–acute kidney injury; PSM = propensity score matching; RR = relative risk. Restricted subgroup excludes patients with septic shock at presentation.

## Data Availability

The data that support the findings of this study were obtained from the TriNetX US Collaborative Research Network. Restrictions apply to the availability of these data, which were used under license for the current study and therefore are not publicly available. Aggregate data and analytic outputs generated within the TriNetX platform are available from the corresponding author upon reasonable request, subject to TriNetX data access policies and institutional permissions.

## Abbreviations

The following abbreviations are used in this manuscript:

AASLD	American Association for the Study of Liver Diseases
AGA	American Gastroenterological Association
AKI	Acute kidney injury
CI	Confidence interval
CKD	Chronic kidney disease
Cox model	Cox proportional hazards model
EASL	European Association for the Study of the Liver
EHR	Electronic health record
HIPAA	Health Insurance Portability and Accountability Act
HR	Hazard ratio
HRS-AKI	Hepatorenal syndrome–acute kidney injury
ICD-10	International Classification of Diseases, Tenth Revision
ICU	Intensive care unit
INR	International normalized ratio
IRB	Institutional Review Board
MELD-Na	Model for End-Stage Liver Disease–Sodium
PSM	Propensity score matching
RD	Risk difference
RR	Relative risk
SBP	Spontaneous bacterial peritonitis
SCCM	Society of Critical Care Medicine
SE	Standard error
SMD	Standardized mean difference
USA	United States of America
